# MetaDBSite: a meta approach to improve protein DNA-binding sites prediction

**DOI:** 10.1186/1752-0509-5-S1-S7

**Published:** 2011-06-20

**Authors:** Jingna Si, Zengming Zhang, Biaoyang Lin, Michael Schroeder, Bingding Huang

**Affiliations:** 1 Systems Biology Division, Zhejiang-California International NanoSystems Institute, Zhejiang University, Kaixuan Road 268, 310029, Hangzhou, China; 2 Bioinformatics Group, Biotechnology Center, Technical University of Dresden, Tatzbergstr 47, 01307, Dresden, Germany; 3 College of Biological Sciences, China Agricultural University, 100193, Beijing, China

## Abstract

**Background:**

Protein-DNA interactions play an important role in many fundamental biological activities such as DNA replication, transcription and repair. Identification of amino acid residues involved in DNA binding site is critical for understanding of the mechanism of gene regulations. In the last decade, there have been a number of computational approaches developed to predict protein-DNA binding sites based on protein sequence and/or structural information.

**Results:**

In this article, we present metaDBSite, a meta web server to predict DNA-binding residues for DNA-binding proteins. MetaDBSite integrates the prediction results from six available online web servers: DISIS, DNABindR, BindN, BindN-rf, DP-Bind and DBS-PRED and it solely uses sequence information of proteins. A large dataset of DNA-binding proteins is constructed from the Protein Data Bank and it serves as a gold-standard benchmark to evaluate the metaDBSite approach and the other six predictors.

**Conclusions:**

The comparison results show that metaDBSite outperforms single individual approach. We believe that metaDBSite will become a useful and integrative tool for protein DNA-binding residues prediction. The MetaDBSite web-server is freely available at http://projects.biotec.tu-dresden.de/metadbsite/ and http://sysbio.zju.edu.cn/metadbsite.

## Background

Protein-DNA complexes perform essential functions in many cellular activities. For example, transcription factors bind to specific DNA sequences in promoters to activate gene expression [1]. Protein-DNA interactions also play important roles in many other biological processes, including DNA replication, DNA repairing, viral infection, DNA packing and DNA modifications [2]. However, the biophysical mechanism of protein-DNA interactions is not clear and the identification of protein-DNA interactions by experimental methods is difficult at present.

Although there are more than 60,000 experimentally determined structures deposited in the current (June 2010) Protein Data Bank database [3] , there are only several hundreds structures on protein-DNA complexes, which is much smaller than the number of protein-DNA complexes in nature. With recent advances in DNA sequencing such as next-generation sequencing technology, genome sequences for many organisms were completed in recent years, producing a huge amount of protein sequences, many of which are DNA-binding proteins. Predicting the DNA binding properties of these DNA binding proteins will be very useful in helping understanding their biological functions.

There are several state-of-the-art prediction servers for predicting DNA bindings based on protein sequences, including DISIS [2], DNABindR [4], BindN [5], BindN-rf [6], DP-Bind [7] and DBS-PRED [8]. Table 1 summarizes the detailed characteristics of these six servers. These six web servers are all based on protein sequences and they combined several features derived from sequence information, such as amino acid frequency, evolutionary profile, sequence conservation, predicted secondary structure, predicted solvent accessibility, electrostatic potential, hydrophobicity, BLOSUM62 matrix, position-specific scoring matrix (PSSM) etc [2,4,5,7]. Furthermore, various machine learning methods are used in these servers, including support vector machine (SVM) [9], Naïve Bayes classifier, random forest [10] and neural network [11].

**Table 1:** Summary of detailed characteristics of the six available web servers for DNA-binding sites prediction.

	Machine learning methods	Properties used in training	Online website
DISIS	Support Vector Machine (SVM) Neural network	Evolutionary profile Conservation Predicted secondary structure Predicted solvent accessibility	http://cubic.bioc.columbia.edu/services/disis

DNABindR	Naïve Bayes classifier	Relative solvent accessibility Sequence entropy Secondary structure Electrostatic potential Hydrophobicity	http://turing.cs.iastate.edu/PredDNA/predict.html

BindN	SVM	The side chain pKa value Hydrophobicity index Molecular mass	http://bioinfo.ggc.org/bindn/

BindN-rf	Random forest	The side chain pKa value Hydrophobicity index Molecular mass Blast-based conservation Biochemical feature Position-specific scoring matrix (PSSM)	http://bioinfo.ggc.org/bindn-rf/

DP-Bind	SVM Kernel logistic regression Penalized logistic regression	Sequence-based BLOSUM62 PSSM-based	http://lcg.rit.albany.edu/dp-bind/

DBS-PRED	Neural network	Protein sequence information Solvent accessibility Secondary structure	http://www.netasa.org/dbs-pred

However, several limitations impair the application of the above servers: each method constructed their own dataset; had their own definition of binding sites; used different parameters derived from sequences; applied different machine learning methods, produced different accuracy and sensitivity, and calculated at much different speed. Therefore, a better and more consistent prediction server is needed. To meet this goal, we have developed metaDBSite, a meta web server for predicting protein DNA-binding sites based solely on amino acid sequences of proteins. MetaDBSite combined the six available online web servers mentioned in Table 1. MetaDBSite used support vector machine (SVM) learning method to learn and test the data. We constructed a large dataset PDNA-316 from PDB and compared the performance of MetaDBSite and the six servers. We showed that the MetaDBSite has a higher sensitivity in distinguishing DNA binding sites on the benchmark dataset. We believe that metaDBSite will become a useful tool for predicting protein-DNA binding residues for relevant researchers.

## Results and discussion

### Performance on PDNA-316 benchmark dataset

**Figure 1:**
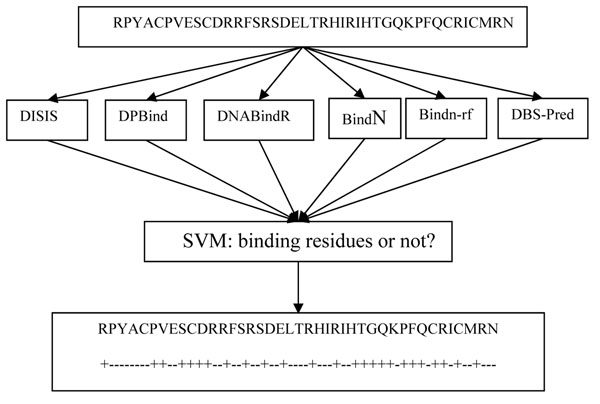
The prediction workflow of the metaDBSite approach. The protein sequence is submitted to the six predictors and the prediction results are retrieved. Then these predicted results are input into the trained SVM and the final prediction (which residues are DNA-binding sites (marked as “+”) is made.

The detailed procedure of metaDBSite is illustrated in Figure 1. Table 2 shows the prediction results for metaDBSite approach (10-fold SVM cross-validation) and the other six methods alone, on PDNA-316 benchmark dataset. It is noted that DISIS gained 19% sensitivity but with very high accuracy and specificity. It is also noted that DISIS failed to return any prediction for over 60 proteins in this dataset due to the strict restriction in its web-server parameters. In such a case, small binding sites with very high confidence were found. However, in the same time, many real DNA-binding residues were missing. In a prediction, the balance of exact value and confident level is important. Therefore, the DISIS method, with high accuracy and specificity but low sensitivity, is incomparable with the other methods. By integrating the prediction results from the six methods, MetaDBSite has achieved 77% sensitivity value and it is much higher than each single method. This sensitivity is 8 percentages higher than that of DPBind method, which has the highest sensitivity among the single methods. Moreover, the strength of metaDBSite is 77%, which also holds the line with the best one among the six methods. Although the accuracy of metaDBSite is a little lower than some of the single methods, metaDBSite is still considered to be the best because of the best performance of sensitivity and strength. Sensitivity is the measurement of DNA-binding residues prediction, which is the most interest point for relevant researchers. Strength is considered to be fair evaluation criteria when the datasets are imbalanced in previous studies [8,12]. In such cases, sensitivity and strength of metaDBSite are also better than each single method; especially sensitivity has gained an obvious improvement. For the other two measurements MCC and F-Measure, metaDBSite has got similar values with the best single method. Overall, metaDBSite outperforms each single method and it also provides the users some analysis and comparison among different methods.

**Table 2:** The prediction results of metaDBSite (10-fold SVM cross-validation) and the other six methods alone for the PDNA-316 benchmark dataset.

Method	Accuracy	Sensitivity	Specificity	Strength	MCC	F-measure
metaDBSite	0.77	0.77	0.77	0.77	0.32	0.33
BindN	0.78	0.54	0.80	0.67	0.21	0.26
BindN-rf	0.82	0.67	0.83	0.75	0.32	0.34
DBS-PRED	0.75	0.53	0.76	0.65	0.17	0.23
DISIS	0.92	0.19	0.98	0.59	0.25	0.27
DNABindR	0.73	0.66	0.74	0.70	0.23	0.26
dpBind	0.78	0.69	0.79	0.74	0.29	0.31

### Comparison of various definitions of DNA-binding sites

**Figure 2:**
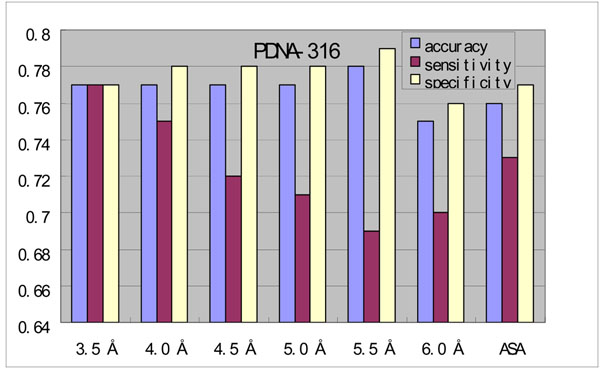
Performance of metaDBSite with DNA-binding site definitions using different distance cut-offs and ASA method on the PDNA-316 benchmark dataset.

In the previous studies, protein-DNA binding sites were defined as those protein residues within a certain distance threshold to the DNA molecule bound to it. The distance thresholds used previously were various, ranging from 3.5 Å to 6.0 Å [8,13-15]. In order to figure out the most proper distance threshold, we have tried several distances in this work. On the other hand, we also defined the DNA-binding sites by solvent accessible surface (SAS) area, *ie*, those residues lost a least 1% SAS area when DNA molecule binds to protein. Figure 2 shows the overall prediction performance of metaDBSite with different definitions on the PDNA-316 dataset. The sensitivity decreased obviously and successively when the distance threshold increased. The accuracy at 3.5 Å distance was just lower than that at 5.5 Å distance. However, the sensitivity at 5.5 Å was 69%, which was much lower than that of 77% at 3.5 Å. The specificity had similar tendency. The specificity in 3.5 Å was not the highest. Therefore, after considering the overall performance of these three measurements together, we chose 3.5 Å as the distance threshold to define the real DNA-binding residues in this study.

### Representative example

**Figure 3:**
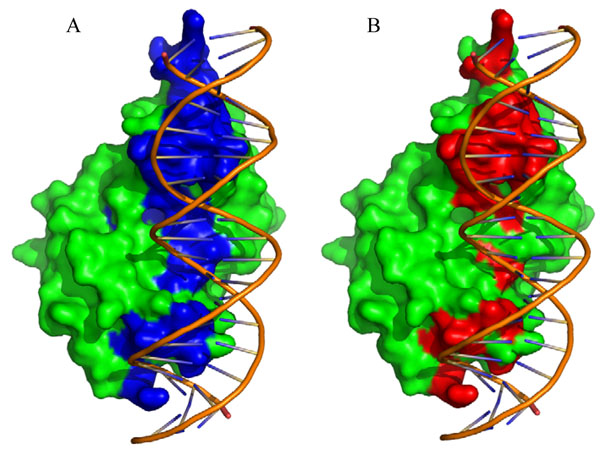
Representative protein-DNA complex: replication initiation protein and its binding DNA regions (PDB ID: 1REP). A: Predicted DNA-binding residues are in blue. B: The real DNA-binding residues defined with 3.5 Å are in red. The replication initiation protein is shown in green.

MetaDBSite reveals its advantage in distinguishing DNA-binding residues sufficiently. In our test dataset, more than 100 proteins were spotted with the sensitivity value of 1.0, which means all the real DNA-binding residues are recognized correctly by metaDBSite. Figure 3 shows an example of these proteins (PDB ID: 1REP, Chain: C). In Figure 3A, those residues in blue are the predicted DNA-binding residues by metaDBSite. In Figure 3B, those residues in red are the real DNA-binding residues defined with 3.5 Å distance threshold. The difference between residues in red and in blue can be seen directly from Figure 3, which is the false positive in the prediction. Here in this protein, the prediction accuracy is 89% and specificity is 88% while sensitivity is 1.0.

### Using structural information to eliminate false positives

It is noted that those six methods and metaDBSite are all using the information of protein sequence only. Since the dataset of PDNA-316 is derived from PDB and the structures for all proteins are known. This structural information of proteins could be used to improve the prediction of metaDBSite. To do this, we used spatial clustering based on the coordinates of the CA atoms of those predicted residues in metaDBSite in the next step, trying to eliminate those false positive predictions (FP). After clustering, those clusters with small number of residues are treated as false positive and thus are removed. We have tried several different parameters in this clustering procedure. All the results have shown that the accuracy and specificity are both increased but the sensitivity decreased (data not shown). In the protein-DNA complex structure, because of the spiral of the DNA molecule, the real DNA-binding residues defined with a distance cut-off do not tend to gather together spatially. Some of the real DNA-binding sites can be 3 or less residues and may locate at an isolated position on protein surface. Therefore, when we eliminate those small clusters, some of TPs may also be removed with FPs at the same time. And this is the reason why we can increase specificity and decrease sensitivity after this clustering post-process.

## Conclusions

DNA-binding residues prediction from protein sequence is of great importance to understand the mechanism of protein-DNA interactions. There have been a lot of research efforts done to discriminate DNA-binding residues from non DNA-binding ones. Various machine learning methods have been applied and different kinds of features based on protein sequence and/or structural information have been used. However, it is hard to directly compare these existing prediction methods because of different data-sets, definitions and evaluation criteria being used. Here, based on the prediction results from six available predictors, we have developed metaDBSite: a meta server for DNA-binding residues prediction based on protein sequence. We evaluated metaDBSite and other 6 predictors on a large data-set using the same definition and criteria. We have shown that MetaDBSite can achieve a better balance of sensitivity and specificity.

**Figure 4:**
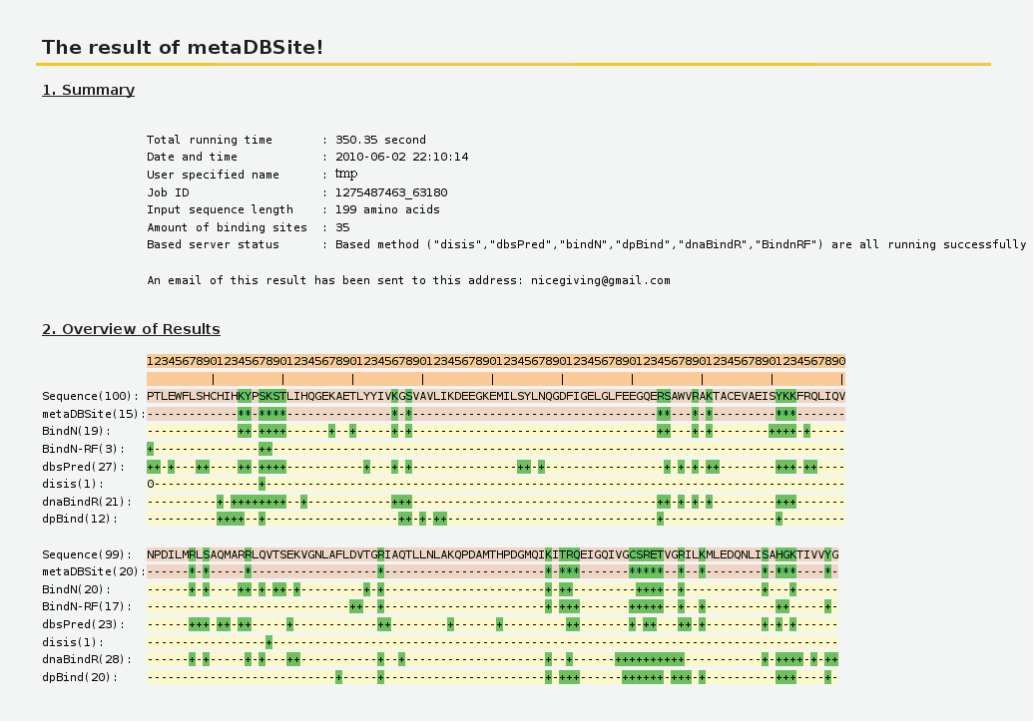
Screenshot of result page on the metaDBSite web-server. The predicted DNA-binding residues are marked “+” for the sixe predictors and “*” for metaDBSite and are all colored green. The non DNA-binding residues are marked “-”.

MetaDBSite is freely available at http://projects.biotec.tu-dresden.de/metadbsite and http://sysbio.zju.edu.cn/metadbsite. The users can simply submit a protein sequence for DNA-binding residues prediction. MetaDBSite will re-direct the submitted sequence to the six predictors automatically and the prediction results are retrieved and analyzed. After the process is finished, the users will be notified by e-mail with a URL to view the prediction results. Figure 4 shows a screenshot of the result page of metaDBSite server. It lists the predicted DNA-binding sites (marked as “*” and “+”) for metaDBSite approach and the other 6 predictors. The whole process for each query normally takes no more than 10 minutes with parallel computational processes on a Linux desktop with a CPU of 2.85 HZ and 2 G memory. If any servers fail to return any prediction due to network problem or server shut-down, metaDBSite will automatically ignore them and continue with those successful predictions.

## Materials and methods

### Benchmark dataset

To evaluate these prediction methods, we derived a large dataset of protein-DNA complexes from current PDB [3]. 865 protein-DNA complexes with resolution better than 3.0 Å were downloaded from PDB and the sequences were submitted to the program H-CD-HIT [16] to get a non-redundant dataset. These 865 proteins are first clustered at a high identity (90%), and then the non-redundant sequences are further clustered at a low identity (60%). A third cluster is performed at lower identity (30%). Default clustering parameters were selected in H-CD-HIT. After clustering, we have 316 protein-DNA complexes in total and it is called PDNA-316 dataset. This dataset is listed in the supplemental data on our metaDBSite web-server and can be downloaded freely.

### Defining real DNA-binding sites

Several previous studies on protein-DNA binding site prediction [8,13-15] have used various definitions of DNA-binding sites. In a protein-DNA complex, an amino acid residue in the protein is defined as DNA-binding site if the distance between any atoms of this residue and any atoms of the DNA molecule is less than a distance threshold. This threshold ranged from 3.5 Å to 6.0 Å in the previous studies. The other residues are regarded as non DNA-binding sites. On the other hand, we also tried to define binding sites with solvent accessible surface area (ASA). We calculated surface area for each protein residue when DNA molecule was absent and present, respectively. The solvent accessible surface area of residues which change at least 1 A^2^ before and after DNA molecule appeared are considered to be DNA-binding residues, the other residues are regarded as non DNA-binding residues. In the final metaDBSite approach, distance 3.5 Å was chosen to define the real DNA-binding sites.

### Performance measures

Four performance measures were used in MetaDBSite, which are accuracy, sensitivity, specificity, strength, Mathews correlation coefficient (MCC) and F-measure. They are defined below:

In the formulas above, TP is the abbreviation of true positives (residues predicted to be DNA-binding residues that are in fact DNA-binding residues); TN is the abbreviation of true negatives (residues predicted to be non DNA-binding residues that are in fact non DNA-binding residues); FP is the abbreviation of false positives (residues predicted to be DNA-binding residues that are in fact non DNA-binding residues)); FN is the abbreviation of false negatives (residues predicted to be non DNA-binding residues that are in fact DNA-binding residues). These definitions and measures are comparable to the previous studies.

### SVM learning

In this work, the six predictors were combined into a prediction system called metaDBSite with the assistance of the Support Vector Machine (SVM). As a machine-learning method as a classifier for two classes, SVM aims to find a rule that put each member in a training set into the corresponding class correctly. Here, the SVM was trained to distinguish DNA-binding residues from non-binding residues. DNA binding amino acids were regarded to be positive samples, and non-DNA binding amino acids were considered to be negative samples. The residue was defined as binding site if the distance between any atoms of this residue and any atoms of the DNA molecule was less than 3.5 Å. With this definition, there are 5342 positive samples and 67396 negative samples in the PDNA-316 dataset.

The detailed procedure of metaDBSite is illustrated in Figure 1. The given sequence is submitted to six web servers and the prediction results are retrieved. Among these six predictors, four of them (i.e., DISIS, DNABindR, BindN, and BindN-rf) return the prediction based on their own scoring functions. The residues with a score above a certain threshold are considered as DNA-binding residues. These scores provide us four input parameters for SVM. For the other two predictors: DP-Bind and DBS-PRED, they only indicate which residues are predicted to bind to DNA or not. Therefore, we simply add a score “+1” to binding sites and “0” to non-binding sites in these two methods. Finally, a total of six parameters are used in the SVM training.

The PDNA-316 datasets were divided into 10 roughly equal subsets. 10-fold cross-validation was performed here. To predict whether a given amino acid in a sequence belongs to the DNA binding site or non-DNA binding site, the subset to which this residue belongs was labelled as the “test” set, whereas the nine remaining subsets were labelled as “training” sets. SVM models were developed for each of the “training” sets. The class label for positive and negative samples was set to +1 and -1, respectively. The ratio of positive to negative samples was about 1:10 in the training set. Using the training set at such a ratio would inevitably cause the SVM model to predict every pair as a negative case. The optimized ratio in the training set was set at 1:1. Each training set was modified by discarding a random selection of the negative samples prior to training. The implemented SVM algorithm was LIB-SVM (http://www.csie.ntu.edu.tw/~cjlin/). The applied kernel function was the radial basis function (RBF). The corresponding parameter settings of SVM learning were automatically optimized by LIB-SVM.

## Competing interests

The authors declare that they have no competing interests.

## Authors' contributions

JNS collected the data, implemented codes, and drafted the manuscript. ZZ helped implementation and developed the web server. BH directed the research and seriously revised the manuscript. BL and MS co-supervised the work and revised the manuscript. All authors read and approved the final manuscript.
